# Effectiveness of a Female Community Health Volunteer–Delivered Intervention in Reducing Blood Glucose Among Adults With Type 2 Diabetes: An Open-Label, Cluster Randomized Clinical Trial

**DOI:** 10.1001/jamanetworkopen.2020.35799

**Published:** 2021-02-01

**Authors:** Bishal Gyawali, Rajan Sharma, Shiva Raj Mishra, Dinesh Neupane, Abhinav Vaidya, Annelli Sandbæk, Per Kallestrup

**Affiliations:** 1 Section of Global Health, Department of Public Health, University of Copenhagen, Copenhagen, Denmark; 2 Community Health Development Nepal, Kathmandu, Nepal; 3 Macquarie University Centre for the Health Economy, Macquarie Park, Sydney, Australia; 4 Nepal Development Society, Chitwan, Nepal; 5 Department of Epidemiology, Welch Center for Prevention, Epidemiology, and Clinical Research, Johns Hopkins Bloomberg School of Public Health, Baltimore, Maryland; 6 Department of Community Medicine, Kathmandu Medical College and Teaching Hospital, Kathmandu, Nepal; 7 Department of Public Health, Aarhus University, Aarhus, Denmark; 8 Steno Diabetes Centre Aarhus, Aarhus, Denmark

## Abstract

**Importance:**

Female community health volunteers (FCHVs) are frontline community health workers who have been a valuable resource in improving public health outcomes in Nepal, but their value is understudied in diabetes care.

**Objective:**

To assess whether an FCHV-delivered intervention is associated with reduced blood glucose levels among adults with type 2 diabetes.

**Design, Setting, and Participants:**

This community-based, open-label, 2-group, cluster randomized clinical trial with a 12-month delayed control group design was conducted in 14 clusters of a semiurban setting in Western Nepal. A total of 244 adults with type 2 diabetes were recruited between November 2016 and April 2017. The follow-up assessment was conducted at 12 months after enrollment. Data analysis was performed from January to February 2019.

**Interventions:**

Seven clusters were randomized to the FCHV-delivered intervention in which 20 FCHVs provided home visits 3 times a year (once every 4 months) for health promotion counseling and blood glucose monitoring. If participants had blood glucose levels of 126 mg/dL or higher, the FCHVs referred them to the nearest health facility, and if participants were taking antihyperglycemic medication, they were followed up by the FCHVs for adherence to their medication. Seven clusters were randomized to usual care (control group).

**Main Outcomes and Measures:**

The primary outcome was the change in mean fasting blood glucose from baseline to 12-month follow-up. Secondary outcomes included changes in mean systolic blood pressure, mean diastolic blood pressure, mean body mass index, percentage change in the proportion of low physical activity, harmful alcohol consumption, current smoking, low fruit and vegetable intake, and antihyperglycemic medication status.

**Results:**

Of 244 participants, 120 women (56.6%) and 92 men (43.4%) completed the trial. At baseline, the mean (SD) age was 51.71 (8.77) years; 127 participants were in the intervention group, and 117 participants were in the control group (usual care). At baseline, the mean (SD) fasting blood glucose level was 156.06 (44.48) mg/dL (158.48 [45.50] mg/dL in the intervention group and 153.43 [43.39] mg/dL in the control group). At 12-month follow-up, the mean fasting blood glucose decreased by 22.86 mg/dL in the intervention group, whereas it increased by 7.38 mg/dL in the control group. The mean reduction was 27.90 mg/dL greater with the intervention (95% CI, −37.62 to −18.18 mg/dL; *P* < .001). In secondary outcome analyses, there was a greater decline in mean systolic blood pressure in the intervention group than in the control group (−5.40 mm Hg; 95% CI, −8.88 to −1.92 mm Hg; *P* = .002). There was detectable difference in the intake of antihyperglycemic medication between the groups (relative risk, 1.35; 95% CI, 1.1 to 1.74; *P* = .02).

**Conclusions and Relevance:**

These findings suggest that an FCHV-delivered intervention is associated with reduced blood glucose levels among adults with type 2 diabetes in a low-resource setting in Nepal.

**Trial Registration:**

ClinicalTrials.gov Identifier: NCT03304158

## Key Points

**Question:**

Is a female community health volunteer (FCHV)–delivered intervention associated with reduced blood glucose levels among adults with type 2 diabetes?

**Findings:**

In this cluster randomized clinical trial involving 244 adults with type 2 diabetes, the FCHV-delivered intervention was associated with a significant reduction in fasting blood glucose level (−27.90 mg/dL) compared with the control group over 12 months.

**Meaning:**

These findings suggest that interventions delivered by FCHVs could improve diabetes control among adults with type 2 diabetes in a low-resource setting.

## Teaser

This cluster randomized clinical trial examines whether an intervention delivered by female community health volunteers was more effective than usual care in reducing blood glucose among adults with type 2 diabetes in Nepal.

## Introduction

Nepal, a developing country in South Asia, is currently experiencing an epidemic of diabetes as a result of epidemiologic and demographic transitions that have impacted lifestyle changes. A systematic review and meta-analysis^1^ of 10 studies involving 30 218 adults estimated the prevalence of type 2 diabetes to be 8.4%. Despite the large burden of type 2 diabetes in Nepal, awareness of diabetes control is poor,^2^ and effective strategies to manage this disease are urgently warranted.

Control of type 2 diabetes is associated with reductions in diabetes complications and premature deaths, thus reducing the burden of the disease. This requires that individuals with diabetes incorporate various interventions, including adherence to medications, regular measurement of blood glucose level, diet, and physical activity.^3^ However, Nepal’s health system has serious deficits in terms of its ability to cope with the growing prevalence of diabetes.^4^ A nationally representative survey^5^ from Nepal reported that more than 50% of adults with diagnosed diabetes did not take their antihyperglycemic medications, whereas 89% did not monitor their blood glucose. Furthermore, approximately 80% of those receiving diabetes treatment had uncontrolled blood glucose levels.^6^

Recent reviews^7,8^ have suggested that a community-based approach to diabetes involving community health workers (CHWs) has immense potential for improving diabetes care and outcome. A few existing studies showing positive outcomes have had several methodological limitations in reporting results, including the lack of true randomization,^9^ a short follow-up period, and the lack of a control group.^10^ Moreover, CHWs served as a part of a multidisciplinary intervention team with doctors, pharmacists, or specialist nurses providing specialist care and support.^7^ This diversity makes it challenging to draw definite conclusions about the independent effect of CHW interventions. Thus, more research using rigorous study design is required to empirically establish the role of CHWs in diabetes management.

Nepal started involving CHWs—namely, female community health volunteers (FCHVs)—in its health care system in the early 1980s.^11^ The FCHVs are local women who serve voluntarily within the government system in all areas of Nepal. They are recruited through mothers’ groups (ie, women’s groups active in different local social and health activities within their locality) and are considered as the first point of contact between community members and primary health care facilities. One strength of the FCHV program is that they perceive being self-empowered through volunteering, which keeps them motivated at work.^12^ Currently, approximately 50 000 FCHVs contribute to critical public health services in Nepal, including in immunization, vitamin A supplementation, and maternal and child health.^13^ Despite FCHVs’ positive record of collaborating with the Nepalese health system, these cadres are yet to be mobilized for the prevention and control of diabetes.

The aim of this study was to assess the effectiveness of involving FCHVs in reducing blood glucose levels in adults with type 2 diabetes in a semiurban setting of Nepal. Our hypothesis was that individuals in the intervention group would see a greater reduction in mean blood glucose level compared with the control group over the succeeding 12 months.

## Methods

The study was approved by the Ethical Review Board of the Nepal Health Research Council . The study protocol is shown in Supplement 1. All participants gave their written informed consent as per the Helsinki Declaration.^14^ The study was conducted following the Consolidated Standards of Reporting Trials (CONSORT) reporting guideline.

### Study Design, Setting, and Participants

This was a community-based, open-label, 2-group, cluster randomized clinical trial with a 12-month delayed control group design in 14 wards (clusters) of the Pokhara Metropolitan City in Nepal, previously named Lekhnath Municipality. Detailed methods of the trial can be found elsewhere.^15^ The randomization was conducted by a biostatistician using a computer-generated sequence to randomly assign (1:1) 14 clusters to the intervention group or control group. Allocation concealment for participants was ensured by selecting them before randomizing wards. Masking of participants and field investigators was not possible because of the preventive nature of the intervention in a community setting.

We adopted the sampling frame from the COBIN trial.^16^ The eligible participants were those from the semiurban area who had previously been involved in a community-based prevalence survey, were aged 25 to 65 years, had a fasting blood glucose level of greater than or equal to 126 mg/dL (to convert to millimoles per liter, multiply by 0.0555) or had already received a diagnosis of type 2 diabetes, and did not intend to migrate outside the study area for at least 12 months.^6^ We excluded individuals with severe illness or pregnancy and those who did not give consent for participation.

The enrollment of participants in the trial occurred between November 2016 and April 2017. Baseline surveys were conducted through face-to-face interviews by 8 trained field investigators with a health professional background. The field investigators conducted interviews in the Nepali language adopting the previously validated World Health Organization STEPwise approach to chronic disease risk factor surveillance tool.^17,18^ The questionnaire elicited sociodemographic information (age, sex, ethnicity, education, marital status, occupation, and income) and behavioral characteristics (fruit and vegetable intake, harmful alcohol consumption, current smoking, physical activity, and receipt of antihyperglycemic medication). Participants’ anthropometric data (height and weight), blood glucose level, and blood pressure were also measured. The follow-up of the trial was conducted at 12 months using the World Health Organization STEPwise tool to measure participants’ anthropometric data, blood glucose level, and blood pressure.

### Interventions

The intervention included a training of FCHVs about type 2 diabetes management and health promotion counseling for prevention of diabetes risk factors. Details regarding the FCHVs intervention and FCHVs selection procedure are described elsewhere.^19^ Briefly, 20 FCHVs from the 7 intervention clusters were trained during a 5-day (approximately 240 minutes per day) interactive training session in a local district hospital. The training imparted knowledge and skills on several topics, such as definition and overview of diabetes, an overview of both modifiable and nonmodifiable risk factors associated with type 2 diabetes, screening procedures of blood glucose level and blood pressure and obtaining body measurements (eg, height and weight), home-based health education and counseling, and effective communication for behavior change. FCHVs were also trained on how to properly record, report, and follow up. The training materials were developed in consultation with key stakeholders and experts, and with guidance from the Health Belief Model^20^ and Social Support Theory.^21^ A recording register was developed and distributed to FCHVs to record dates, times, and activities (eFigure in Supplement 2).

Following the training, FCHVs visited the households of members in the intervention group and provided home-based health education focused on information pertaining to the harmful effects of diabetes risk factors, maintaining ideal body weight, smoking cessation, reduction of alcohol drinking, increased consumption of green leafy vegetables and fresh fruits, low-salt diet, and importance of regular use of antihyperglycemic medication. FCHVs also measured participants’ blood glucose obtained via point-of-care testing (not serum) with a glucometer using finger-stick blood sample, blood pressure, height, and weight. If a participant had an elevated blood glucose level (≥126 mg/dL), FCHVs referred the participant to the nearest health facility, and if the participant was taking antihyperglycemic medication, they were also followed-up for adherence to their medication during the FCHV visit. On average, 1 FCHV followed 6 individuals (range, 2-8 individuals) with diabetes during the course of follow-up. The frequency of FCHV visits was 3 times a year (once every 4 months). One field supervisor was responsible for supervising all 20 FCHVs. A supervision checklist was used to track and update the knowledge and skills of FCHVs (eTable 1 in Supplement 2). FCHVs were reimbursed for transport costs and refreshments (US $5) during the training and for each household visit.

Participants in the control groups continued to manage their diabetes as usual. They did not receive further contact, information, or educational materials from FCHVs. Health education on diabetes management was delivered to the control group in a 3-day workshop held immediately after the follow-up period.

### Outcomes

The primary outcome was the change in mean fasting blood glucose from baseline to 12-month follow-up. Diagnosis of diabetes status was based on fasting blood glucose level following the updated World Health Organization guidelines.^22^ Participants were considered to have type 2 diabetes if a doctor had previously diagnosed it, and/or if they were taking antihyperglycemic medications, and/or had a fasting blood glucose level greater than or equal to 126 mg/dL. The finger-stick method was used to collect capillary blood samples after 8 hours of fasting using a standardized digital glucometer.

Secondary outcome measures included changes in mean systolic blood pressure, mean diastolic blood pressure, mean body mass index (BMI; calculated as weight in kilograms divided by height in meters squared), and percentage change in the proportion of the following risk factors: low physical activity, harmful alcohol consumption, current smoking, low fruit and vegetable intake, and receiving antihyperglycemic medication. Smokers were defined as those who smoked cigarettes or used tobacco variants (*bidi, kankat*, or* hukka*) or other forms of smokeless tobacco daily. We defined harmful alcohol consumption if participants reported having drunk 15 or more standard units of alcohol in a week for men and 8 or more standard units in a week for women.^23^ Participants consuming fewer than 5 servings of fruits or vegetables in a week were categorized as having low fruit and vegetable intake.^24,25^ Anthropometric measurements for calculating BMI included height measured in meters using a portable standard stature scale and weight in kilograms using personal digital scales. Low physical activity was defined as fewer than 3000 metabolic equivalents of tasks of vigorous or moderate activity each week.^26^ Blood pressure was measured using an automated digital blood pressure monitor after the participants had been seated 5 five minutes. Three measurements were made at 5-minute intervals, and the average of the last 2 measurements was used for analysis.

### Statistical Analysis

The study sample size was calculated to detect an 18 mg/dL (1 mmol/L) difference in fasting blood glucose between the intervention and control groups after 12 months of follow-up.^27,28^ Using 2-sided *t* tests (*P* < .05) and assuming an SD of 38 mg/dL^29^ and 80% power, we determined that we would need 7 clusters with 13 individuals per intervention group. We presumed an intraclass correlation coefficient of 0.01,^30^ giving a design effect of 1.12 (1 + 0.01[13 − 1] = 1.12). Allowing for up to 20% loss to follow-up, the sample size was adjusted to 16 participants in each cluster (ie, 112 individuals per group or a total of 224 diabetic individuals). Baseline characteristics between groups were compared using analysis of variance (for continuous variables) and χ^2^ or Fisher exact test (for categorical variables). Primary data were analyzed using the intention-to-treat principle. We used mixed-effects linear regression to model the primary outcome and continuous secondary outcomes. For the secondary outcomes with binary responses, mixed-effects logistic regression analyses were used to estimate relative risk (RR). Random effects were specified for study clusters and for participants to account for the clustered study design. The outcome data for individuals who had dropped out of the study were imputed using multiple imputations by chained equations, pooling results from 10 imputed data sets according to the Rubin rule.^31^ The imputation model included variables related to outcome and study groups and those associated with missing outcome information. We conducted a complete-case analysis, and the results were nearly identical to those that used imputed data. We also performed subgroup analyses for sex, age, and severity of blood glucose to examine their effect size on the mean fasting blood glucose. To explore the heterogeneity of intervention effect by subgroup, an interaction term between the intervention assignment and subgroup was included in the models, and its significance was tested using the Wald test. All *P* values were from 2-sided tests and results were deemed statistically significant at *P* < .05. Data were entered in EpiData statistical software version 3.1 (EpiData Association) and analyzed using Stata statistical software version 14.2 (StataCorp). Data analysis was performed from January to February 2019.

## Results

**Figure 1.**
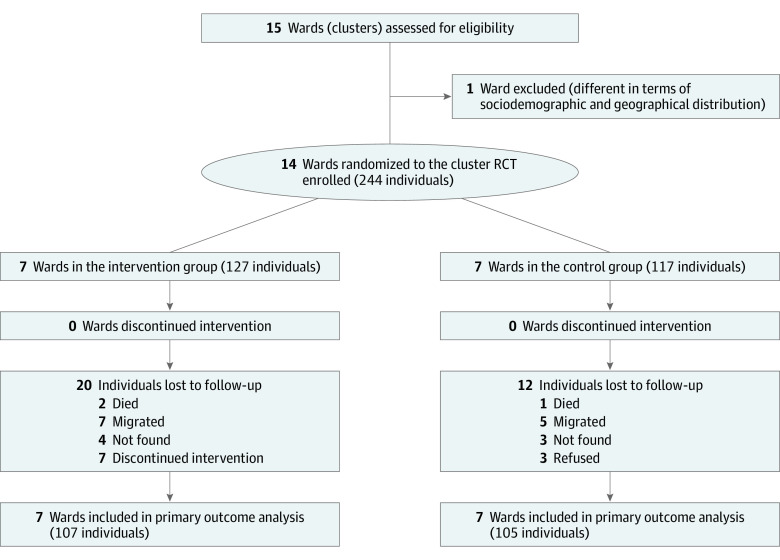
CONSORT Diagram for Cluster Randomized Clinical Trial (RCT) Showing Randomization Allocation, Follow-up, and Analysis

Of 244 participants from 14 clusters, 120 women (56.6%) and 92 men (43.4%) completed the trial. At baseline, the mean (SD) age was 51.71 (8.77) years; 127 participants were in the intervention group and 117 participants were in the control group (usual care) (Figure 1). At baseline, the mean (SD) fasting blood glucose level was 156.06 (44.48) mg/dL (158.48 [45.50] mg/dL in the intervention group and 153.43 [43.39] mg/dL in the control group), mean (SD) systolic blood pressure was 133.74 (18.69) mm Hg, mean (SD) diastolic blood pressure was 85.77 (10.66) mm Hg, 65 participants (26.0%) were current smokers, 34 (14.0%) had harmful alcohol use, and 46 (19.0%) had a low physical activity level. There were no differences in baseline characteristics of participants between the intervention and control groups except for antihyperglycemic medication and family history of diabetes (Table 1 and eTable 2 in Supplement 2).

**Table 1.** Baseline Characteristics of the Intention-to-Treat Sample^a^

Characteristic	Participants, No. (%)	
	Intervention group (n = 127)	Control group (n = 117)
Cluster level, No.		
Wards	7	7
Female community health workers	20	0
Sociodemographic characteristics		
Age, mean (SD), y	51.17 (8.77)	52.29 (8.78)
Sex		
Male	59 (46.5)	44 (37.6)
Female	68 (53.5)	73 (62.4)
Education		
Low	67 (52.8)	64 (54.7)
Medium	43 (33.8)	39 (33.3)
High	17 (13.4)	14 (6.0)
Occupation		
Agriculture	46 (36.2)	34 (29.1)
Employee	25 (19.7)	22 (18.8)
Housemaker	38 (29.9)	44 (37.6)
Labor	3 (2.4)	2 (1.7)
Others	15 (11.8)	15 (12.8)
Monthly household income, mean (SD), Nepalese Rupees^b^	28 169.29 (25 543.15)	31 606.83 (28 633.52)
Marital status		
Married	117 (92.1)	105 (89.7)
Unmarried	10 (7.9)	12 (10.3)
Clinical characteristics, mean (SD)		
Weight	65.17 (11.45)	65.91 (11.52)
Body mass index^c^	26.39 (3.96)	26.84 (4.11)
Systolic blood pressure, mm Hg	134.14 (16.73)	133.30 (20.68)
Diastolic blood pressure, mm Hg	86.00 (8.99)	85.51 (12.25)
Fasting blood glucose, mean (SD), mg/dL	158.48 (45.50)	153.43 (43.39)
Behavioral characteristics		
Current smoking^d^	38 (29.9)	27 (23.1)
Harmful alcohol consumption^e^	21 (16.5)	13 (11.1)
Low physical activity^f^	23 (18.1)	23 (19.7)
Low fruit and vegetables intake^g^	122 (96.1)	114 (97.4)
Medical history		
Family history of diabetes	84 (66.1)	60 (51.3)
Receiving antihyperglycemic medications	73 (57.5)	47 (40.5)

SI conversion factor: To convert blood glucose to mmol/L, multiply by 0.0555.

^a^ All data are unadjusted.

^b^ As of December 22, 2020, 1 Nepalese Rupee = $US 0.0085.

^c^ Body mass index is calculated as weight in kilograms divided by height in meters squared.

^d^ Current smoking was defined as those who smoked cigarettes, tobacco variants (*bidi*, *kankat*, or *hukka*), or those using other forms of smokeless tobacco daily.

^e^ Harmful alcohol consumption was determined by asking the number of standard drinks consumed in the last 30 days. Harmful alcohol was defined as drinking 15 or more standard units of alcohol a week for men and 8 or more standard units a week for women.

^f^ Low physical activity was defined as less than 3000 metabolic equivalents of tasks of vigorous or moderate activity per week.

^g^ Participants consuming fewer than 5 servings of fruits or vegetables a week were categorized as having low fruit and vegetable intake. One serving of vegetables was defined as 1 cup of raw green leafy vegetables, one-half cup of other vegetables (cooked or chopped raw), or one-half cup of vegetable juice. One serving of fruit was defined as 1 medium-sized piece of fruit; one-half cup of chopped, cooked, or canned fruit; or one-half cup of nonartificially flavored fruit juice.

### Primary Outcome

**Figure 2.**
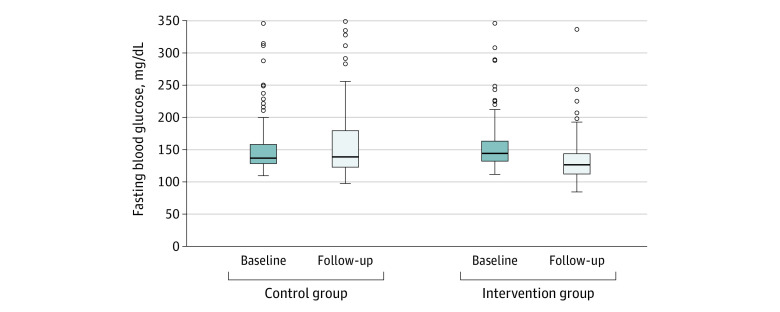
Unadjusted Change in Fasting Blood Glucose From Baseline to Follow-up in Intervention and Control Groups The difference in fasting blood glucose between baseline and follow-up was 7.38 mg/dL (to convert to millimoles per liter, multiply by 0.0555) for the control group and −22.86 mg/dL for the intervention group. Ends of the boxes denote the 25th and 75th percentiles, the horizontal lines inside the boxes denote the means, the error bars denote 95% CIs, and the circles denote outliers.

At 12 months, the mean fasting blood glucose decreased by 22.86 mg/dL in the intervention group, whereas it increased by 7.38 mg/dL in the control group (Figure 2). The mean reduction was 27.90 mg/dL with the intervention (95% CI, −37.62 to −18.18 mg/dL; *P* < .001) (Table 2). This finding was similar in imputation analyses that included those who were not followed up (eTable 3 in Supplement 2).

**Table 2.** Primary and Secondary Outcomes

Outcomes	Intervention group (n = 107)	Control group (n = 105)	Intervention effect size	*P* value^a^
Fasting blood glucose, mean (SD), mg/dL^b^				
At baseline	157.14 (42.66)	153.18 (43.38)	−27.90 (−37.62 to −18.18)^c^	<.001
At 12 mo	134.28 (36.00)	160.56 (55.44)		
Change	−22.86 (38.70)	7.38 (36.00)		
Systolic blood pressure, mean (SD), mm Hg				
At baseline	133.55 (15.36)	132.19 (18.60)	−5.40 (−8.88 to −1.92)^c^	.002
At 12 mo	125.69 (13.76)	130.04 (16.76)		
Change	−7.86 (11.36)	−2.15 (12.28)		
Diastolic blood pressure, mean (SD), mm Hg				
At baseline	85.98 (7.99)	85.29 (11.86)	−1.50 (−4.83 to 1.83)^c^	.37
At 12 mo	80.75 (8.98)	81.69 (9.50)		
Change	−5.23 (7.88)	−3.60 (9.02)		
Body mass index^d^				
At baseline	26.74 (4.00)	27.12 (4.01)	−0.26 (−0.69 to 0.15)^e^	.22
At 12 mo	26.31 (3.90)	27.00 (4.07)		
Change	−0.43 (1.55)	−0.12 (1.26)		
Low physical activity				
At baseline	20 (18.7)	19 (18.1)	1.01 (0.90 to 1.11)^e^	.98
At 12 mo	13 (12.2)	13 (12.4)		
Change	1.5 (0.8 to 2.9)	1.4 (0.7 to 2.8)		
Harmful alcohol consumption				
At baseline	18 (16.8)	11 (10.5)	1.19 (0.61 to 2.34)^e^	.59
At 12 mo	12 (11.2)	12 (11.4)		
Change	1.5 (0.8 to 3.0)	0.9 (0.4 to 1.9)		
Current smoking				
At baseline	30 (28.0)	24 (22.9)	1.23 (0.89 to 1.69)^e^	.20
At 12 mo	32 (29.9)	22 (20.9)		
Change	0.9 (0.6 to 1.4)	1.1 (0.6 to 1.8)		
Low fruit and vegetable intake				
At baseline	102 (95.3)	102 (97.1)	0.97 (0.92 to 1.03)^e^	.41
At 12 mo	102 (95.3)	103 (98.1)		
Change	1.0 (0.9 to 1.1)	1.0 (0.9 to 1.1)		
Receiving antihyperglycemic medications				
At baseline	63 (58.8)	43 (40.9)	1.35 (1.10 to 1.74)^e^	.02
At 12 mo	64 (59.8)	49 (46.6)		
Change	0.9 (0.8 to 1.2)	0.8 (0.6 to 1.2)		

SI conversion factor: To convert blood glucose to mmol/L, multiply by 0.0555.

^a^ For continuous outcomes, *P* values were calculated from linear mixed-effects models with random intercepts for wards and participants. The estimated intervention effect was controlled for age, sex, antihyperglycemic medication status, family history of diabetes, monthly household income, and the baseline summary of the respective outcomes. For binary outcomes, *P* values were calculated from mixed-effects logistic regression analyses with random intercepts for wards and participants. The estimated intervention effect was controlled for age, sex, antihyperglycemic medication status, family history of diabetes, monthly household income, and the baseline summary of the respective outcomes.

^b^ The prespecified primary outcome was the change in mean fasting blood glucose from baseline to 12-month follow-up. Intraclass correlation coefficient from the linear mixed effects model for change in fasting blood glucose was less than 0.001.

^c^ Shown is the difference in mean change and 95% CI.

^d^ Body mass index is calculated as weight in kilograms divided by height in meters squared.

^e^ Shown is the relative risk and 95% CI.

### Secondary Outcomes

**Figure 3.**
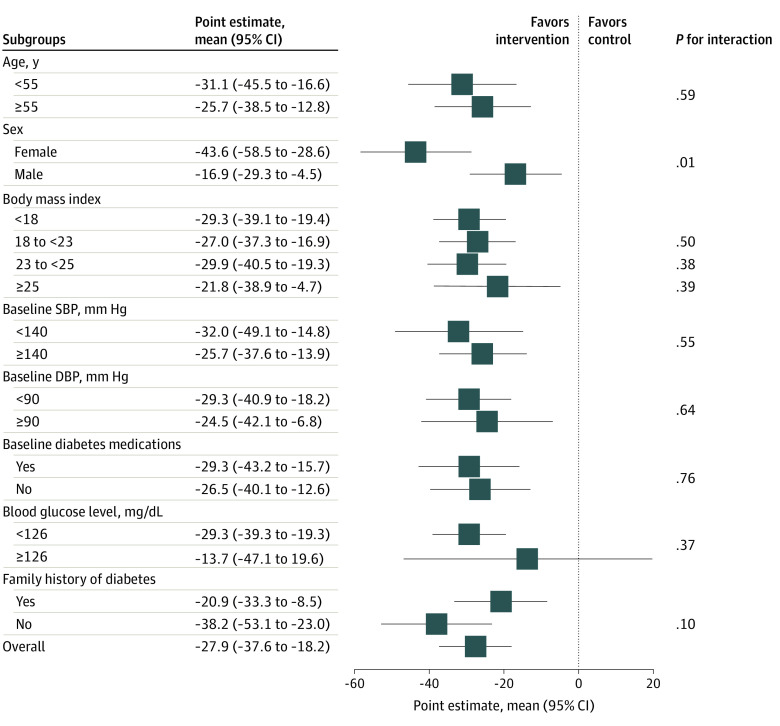
Forest Plot of Differences in Mean Fasting Blood Glucose by Subgroup According to Different Characteristics of the Sample The dashed line represents the line of no effect. Symbols show point estimates, and error bars denote 95% confidence limits. *P* values indicate subgroup interactions (obtained using mixed-effects models). To convert blood glucose to millimoles per liter, multiply by 0.0555. Body mass index is calculated as weight in kilograms divided by height in meters squared. DBP indicates diastolic blood pressure; and SBP, systolic blood pressure.

The change in mean systolic blood pressure was 5.40 mm Hg greater in the intervention group compared with the control group (95% CI, −8.88 mm Hg to −1.92 mm Hg; *P* = .002). There was no evidence for an association of the intervention with diastolic blood pressure (mean change, −1.50 mm Hg; 95% CI, −4.83 mm Hg to 1.83 mm Hg). Similar results were observed for BMI (mean change, −0.26; 95% CI, −0.69 to 0.15) (Table 2). Both intervention and control groups demonstrated an increase in intake of antihyperglycemic medication (RR 1.35; 95% CI, 1.05 to 1.74; *P* = .02). There were no changes for low physical activity (RR 1.01; 95% CI, 0.90 to 1.11), consumption of harmful amounts of alcohol (RR, 1.19; 95% CI, 0.61 to 2.34), smoking daily (RR, 1.23; 95% CI, 0.89 to 1.69), and eating fewer than 5 servings of fruit and vegetables each day (RR, 0.97; 95% CI, 0.92 to 1.03) (Table 2). Similar results were obtained in sensitivity analyses that included those who were recruited but not followed up (eTable 3 in Supplement 2). Results were consistent for the effect of the intervention across different sample characteristics but with greater effect in women (mean change, –43.6 mg/dL; 95% CI, –58.5 to –28.6 mg/dL; *P* = .01) (Figure 3).

## Discussion

To our knowledge, this study is the first community-based research study designed to investigate how to reduce blood glucose levels among adults with type 2 diabetes in Nepal. The findings indicated that an FCHV-delivered intervention was associated with reduced blood glucose levels among adults with type 2 diabetes in a semiurban setting in Nepal.

Our intervention has great potential for reducing blood glucose levels and managing diabetes in low-resource settings. The blood glucose reduction attained was comparable to that observed in previous studies.^32,33,34^ In those studies, the CHW-led diabetes interventions were provided via tailored home visits through face-to-face consultations, group sessions, and telephone contacts. The improvement in blood glucose in the intervention group in the present study may partially be explained by the regular and resultant feedback to participants following FCHVs’ assessment. Individuals with diabetes in the intervention group underwent repeated blood glucose measurements every 4 months by FCHVs and were counseled about behaviors related to antihyperglycemic medication adherence, which might have contributed to the improvement in medication adherence and, thus, blood glucose reduction.

In addition, differences in receiving antihyperglycemic medications were found to be significant in our study. This finding is consistent with the evidence presented in other studies^33,35^ conducted in other low-to-middle income countries involving individuals with diabetes. For individuals who had elevated blood glucose, FCHVs likely increased reminders and clarification on how and/or when to take medications, provided education to reduce fears and increase trust, decreased diabetes-related distress, and assisted in overcoming system-related barriers to obtain medications, as well as referred them to the nearest health facility. Also, given that diabetes could lead to increased social isolation, timely intervention by the FCHVs could have improved mental health and desire for self-improvement, including medication adherence. Increase in intake of antihyperglycemic medications was observed in both intervention and control groups. Intervention contamination could have occurred and contributed to the findings observed in the control group. We did not find significant changes in other secondary outcomes, such as in terms of BMI or self-reported measures of fruits and vegetables intake or physical activity. Some behavioral factors have a longer latency period, and it would take much longer intervention and follow-up periods to show an association that our study did not permit because we only had a 1-year follow-up. In fact, frequent and sustained interventions are expected to yield better results because of the dose-response relationship.^36^

Our study results have important implications. Our intervention was simple in terms of design and logistics and can be integrated into the existing health care system in Nepal, where FCHVs have been part of the primary health care system for the past 3 decades. This cadre of health workers may be promoted to take responsibility for community mobilization and education of communities in relation to diabetes management in Nepal. There is also considerable potential to implement and scale up this intervention in communities with limited financial and health care resources but with a strong network of CHWs.

### Limitations

Our study also has certain limitations. First, we used finger-stick blood glucose estimation instead of venous glucose estimation, which would have been ideal, because of logistic and financial barriers. However, a previous study showed a good correlation between capillary blood glucose and venous plasma estimations.^37^ Second, only fasting blood glucose, without other glycemic indexes (eg, glycated hemoglobin A_1c_) or oral glucose tolerance tests, was used for the diagnosis of type 2 diabetes, which may have underestimated the true diabetes prevalence. Third, although we considered the issues of contamination during the study design, there still might have been some contamination between the clusters, which may have diluted the observed association. Fourth, we did not adjust for multiple comparisons, and given the likelihood of type 1 errors,^38^ changes in secondary outcomes observed should be interpreted carefully. Fifth, our intervention was not designed to assess which aspect of the intervention caused a beneficial effect, although we found several indications from our risk-factor analyses.

## Conclusions

The findings of this randomized clinical trial suggest that an FCHV-delivered intervention could be associated with reduced blood glucose level in adults with type 2 diabetes in a low-resource setting in Nepal. Future programs should incorporate measuring medication (amount, start date, and type) and adherence, changes to lifestyle behaviors, and knowledge of diabetes to find out which factors are best targeted to improve the control of type 2 diabetes in these settings.

## Notes

Supplement 1. **Trial Protocol**

Supplement 2. **eFigure. **Recording Register Example **eTable 1. **Supervision Checklist Example **eTable 2. **Baseline Characteristics of the Per Protocol Sample **eTable 3. **Imputation Analysis Using Intention-to-Treat Principles of Changes in Primary and Secondary Outcomes at 12 Months

Supplement 3. **Data Sharing Statement**
