# Supplementary material for: Effectiveness of a Female Community Health Volunteer–Delivered Intervention in Reducing Blood Glucose Among Adults With Type 2 Diabetes: An Open-Label, Cluster Randomized Clinical Trial
Source: JAMA Netw Open. 2021 Feb 1;4(2):e2035799. doi: 10.1001/jamanetworkopen.2020.35799 (PMC7851734; doi:10.1001/jamanetworkopen.2020.35799)
Supplement: Supplement 1. — Trial Protocol [file jamanetwopen-e2035799-s001.pdf]

Research Proposal Approval Format

Research Title: Community Based Management of Diabetes in Nepal: Study Protocol for a Cluster-Randomized Controlled Trial

Nepal Health Research Council (NHRC)

P.O. Box: 7626, Ramshah Path, Kathmandu, Nepal

Tel: +977-1-4254220, 4227460, Fax: +977-1-4262469

E-mail: nhrc@nhrc.org.np, Website: http://www.nhrc.org.np

For Official Use Only

(Please see the check list before Registration of the application form)

Registration No.:

Registration Date:

Approved Date:

Name of PI:

Total Budget of the Project:

NHRC Processing Fee:

Research Site:

Tentative Date of Initiating the Project:

Duration of the Research Project:

Name of Internal Reviewer:

Name of External Reviewer:

Signature & Seal of NHRC:

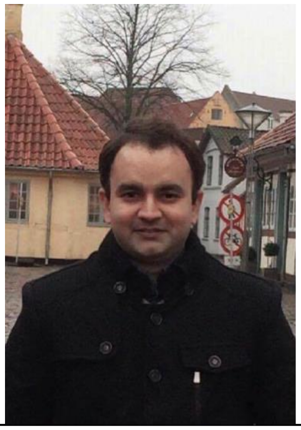

Part – I

Administrative Information

1. Research Title: Community Based Management of Diabetes in Nepal: Study Protocol for a Cluster-Randomized Controlled Trial

2. Name and Title of Principal Investigator responsible for the proposed research:

Gyawali		Bishal
Last (Surname)	Middle (if any)	First name

Nationality: Nepali

Citizenship Number with district name from where it was obtained (only for Nepali) 7193/961 Kathmandu

Passport Number (only for non Nepali citizen):

Signature: Date: 19 August 2016

Postal Address: Bartholins Allé 2, Building 1261, Aarhus University, Aarhus 8000 C, Denmark

Telephone No.:

Mobile No.: +45-42348030

Fax No.:

e-mail: bishalforu@hotmail.com

Alternate e-mail: bishal@ph.au.dk

3. Full name of the Institution associated with the Principal Investigator (if applicable) : Center for Global Health, Department of Public Health, Aarhus University

   Designation: PhD student

   Postal Address (if different from the address given above):

   Telephone No.: +45-42348030

   Fax No.:

   e-mail:

   Website: www.au.dk

4. Declaration of the head of the Institution (if applicable)

   If the proposed research is approved, we will allow him/her to conduct the research in this institution.

   Signature: Date:

Last (Surname)	Middle (if any)	First name
Ledder		Loni

   Designation: Head of Department

   Name of the Institution: Department of Public Health

   Contact/Postal Address: Bartholins Allé 2, building, room 332, 8000 Aarhus C, Denmark

   Telephone No.: +45-20467030

   Fax No.:

   Institutional e-mail: loni.ledderer@ph.au.dk

   Website: www.au.dk

5. Name and Title of Co-investigators responsible for the proposed research (Use the similar format if more than one):

Kallestrup		Per

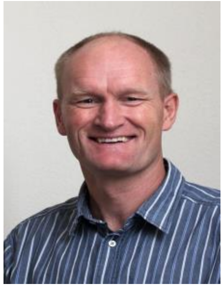

Last (Surname) | Middle (if any) | First name

Nationality: Danish

Citizenship Number with district name from where it was obtained (only for Nepali):

Passport Number (only for non Nepali citizen): 206820507

Affiliated Institution (if applicable): Department of Public Health, Aarhus University

Designation: Associate Professor

Signature:

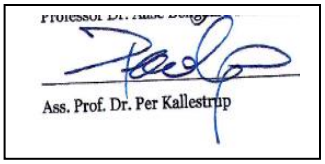

Date: 19.08.2016

Postal Address (if different from the address given above):

Telephone No.: +45-20 926 528

Fax No.:

e-mail: kallestrup@dadlnet.dk

6. Name and Title of Co-investigators responsible for the proposed research (Use the similar format if more than one):

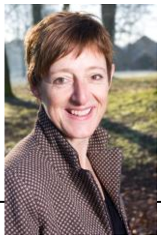

Last (Surname)	Middle (if any)	First name
Sandbæk		Annelli

Nationality: Danish

Citizenship Number with district name from where it was obtained (only for Nepali):

Passport Number (only for non Nepali citizen):

Affiliated Institution (if applicable): Department of Public Health, Aarhus University

Designation: Professor

Signature: Date: 19.08.2016

Postal Address (if different from the address given above):

Telephone No.: +45 21 282 073

Fax No.:

e-mail: annelli.sandbaek@ph.au.dk

7. Name and Title of Co-investigators responsible for the proposed research (Use the similar format if more than one):

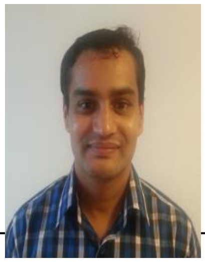

Neupane		Dinesh
Last (Surname)	Middle (if any)	First name

Nationality: Nepali

Citizenship Number with district name from where it was obtained (only for Nepali) 127836 Kaski

Passport Number (only for non Nepali citizen):

Affiliated Institution (if applicable): Department of Public Health, Aarhus University

Designation: PhD student

Signature: Date: 19.08.2016

Postal Address (if different from the address given above):

Telephone No.: +45-20 926 528

Fax No.:

8. Name and Title of Co-investigators responsible for the proposed research (Use the similar format if more than one):

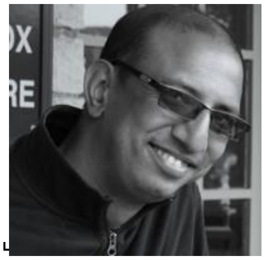

Last (Surname)	Middle (if any)	First name
Vaidya		Abhinav

Nationality: Nepali

Citizenship Number with district name from where it was obtained (only for Nepali)

Passport Number (only for non Nepali citizen):

Affiliated Institution (if applicable): Kathmandu Medical College

Designation: Associate Professor

Signature: Date: 19.08.2016

Postal Address (if different from the address given above):

Telephone No.: +977-9841484422

Fax No.:

e-mail: dr.abhinavvaidya@gmail.com

9. List the name(s) and institutional affiliation to the researcher(s) (other than co-investigator) to assist your project in Nepal and abroad (if any)

Name	*Institution and Address*
(a) Sagar Dahal, District Public Health Office	Kaski
(b) Ram Chandra Paudel, Executive Officer	Lekhnath Municipality

(Use additional sheet if necessary)

10. List the name(s) of Nepali researcher(s) (other than co-investigator) or Nepalese Institution/hospital/NGO(s) etc. from whom you may seek co-operation (if any)

(a) Nepal Development Society

(b) Nepal Health Research Council, Ministry of Health and Population, Nepal

(Use additional sheet if necessary)

8. List major equipment(s) in relation to your research project you plan to bring/import to Nepal (If applicable)

(a) Glucometers, Lancets, Test strips

(b)

(Use additional sheet if necessary)

8.1 List details of all specimen(s) (if any) that you may transport from Nepal in relation to your research.

(a) Digital Data

(b)

(c)

(d)

8.2 Country of Destination: Denmark

Name of Institution: Aarhus University

8.3 Mode of Transportation of Specimen: Electronic device: Computer

8.4 How will you ensure duplicate specimens remain in the country?

Both hard copy and electronic copy will be stored at Nepal Development Society

(If necessary use additional sheet)

9. Is this research part of your Thesis?

Yes [√] No [ ]

If yes,

For what degree and in which subject? PhD in Public Health

From which university? Aarhus University

From which country? Denmark

Part – II

Financial Information

10. Research Title:

Community Based Management of Diabetes in Nepal: Study Protocol for a Cluster-Randomized Controlled Trial

11. Name of the funding organization:

Partial funding from Aarhus University. The project implementation funds are sought from other donors.

Contact information of funding organization or agency:

Postal Address: Nordre Ringgade 1, DK-8000 Aarhus C

Telephone No.: +45 8715 0000

Fax No.:

e-mail:

Contact person at the funding organization or agency:

Kallestrup		Per
Last (Surname)	Middle (if any)	First name

Designation: Associate Professor

Total amount of funds (in NRs / US $) allocated for the proposed research project: US $ 4200

Itemized budget (in detail) and justify the resources required for the proposed research work (*use additional sheet*)

Part – III

Research Proposal Description

12. Research Title:

Community Based Management of Diabetes in Nepal: Study Protocol for a Cluster-Randomized Controlled Trial

13. Proposal Summary (maximum 500 words):

Background: Type 2 diabetes is one of the fastest growing chronic diseases worldwide. Despite its growing burden there is a lack of population approaches to reducing blood sugar among persons with diabetes, especially in developing countries. The health complications associated with type 2 diabetes can be prevented, delayed, or improved via early diagnosis and effective management. This research aims to examine the impact of a family-based home health education intervention on type 2 diabetes provided by Female Community Health Volunteers (FCHVs) at Lekhnath Municipality of Nepal.

Method/design: This is a community-based, open-masked, two-armed, cluster-randomized controlled trial with 7 randomly selected intervention and 7 wait-list control sites in Lekhnath Municipality of Nepal. A total of 112 subjects with type 2 diabetes will be recruited from the intervention sites and 112 subjects from the wait-list control sites. A 12-month family-based life style intervention will be administered through the help of FCHVs based on the Health Belief Model framework. Wards in the wait-list control will continue to manage their poor glycemic condition as usual and their intervention will be delayed for 12 months. Participants will be measured at baseline and 12 months later. The primary outcome is measures of glycemic control mean fasting blood glucose levels. Secondary outcome measures of the study include body mass index, blood pressure, alcohol use, tobacco use, diet intake, knowledge, quality of life, depression scores, physical activity, social support, and sedentary behaviors.

Discussion: The perspective of this study is to develop and implement, in collaboration with the community people, community-based, culturally sensitive diabetes prevention and control programs. Monitoring blood glucose with point-of-care testing at home can diagnose as well as monitor how well diabetes is controlled. Moreover, our study will add to the existing global evidence on models involving lay health workers in the mitigation of the growing burden of non-communicable diseases particularly related to diabetes. If the health promotion package is found to be effective, the approach can be easily adopted into the existing primary health care delivery system of Nepal. It is furthermore anticipated that the model may also serve as an affordable tool for evidence-based integrated care for improvement of diabetes management in other developing countries.

14. Introduction:

14.1 Background of Study (maximum 500 words):

While communicable diseases remain an important public health issue in developing countries, the rising burden of non-communicable diseases and their risk factors are rapidly rising too, posing a double burden on health systems. Diabetes is increasingly becoming a significant public health problem worldwide, leading to high morbidity and mortality resulting from clinically severe complications. The global prevalence of diabetes in 2010 was estimated to be around 6.4%, affecting 285 million adults, and is expected to increase to 7.7% by 2030. With ageing, the prevalence of diabetes is expected to increase by 69% in low-and middle-income countries (1). In particular, type 2 diabetes is becoming more widespread globally, accounting for over 90% of all diabetes cases (2).The rising prevalence is attributed to various modifiable risk factors such as changes in nutrition and life styles leading to physical inactivity and obesity as well as non-modifiable risk factors including family history and age related factors (3). Type 2 diabetes has reached epidemic proportions in South-East Asia Region, leading to significant increases in morbidity and mortality in recent years(4). Indeed, we noted in our previous systematic review and meta-analysis that type 2 diabetes is emerging as a major health care problem in Nepal, with a prevalence rate of 8.4% (5). There is also paucity of data on type 2 diabetes prevalence and available studies are not consistent with in their data collection methods regarding type 2 diabetes and related modifiable risk factors (6). Hence a study of such nature will help us to understand the problem and to design an appropriate intervention for diabetes management at the community level.

14.2 Statement of the Problem and Rationale / Justification (maximum 500 words)

Population-based approaches that reduce the population level of blood glucose by a small amount or that prevent the increase in blood glucose with age can lead to marked reduction in the risk of type 2 diabetes (7). It has been shown that application of population-based interventions to target modifiable preventable risk factors could prevent at least 80% of non-communicable diseases, including type 2 diabetes (8). A number of recent studies offer scientific evidence for reducing the epidemic of type 2 diabetes through support for lifestyle modification(9,10). However, to date prevention programs in low-and middle-income countries have only had a modest effect on reducing diabetes through primary prevention(11) and the best approach to reduce blood glucose level is still unclear. A study by Ramchandran and colleagues in India reported that lifestyle intervention programs followed by advice from a health care advice exhibit a latent potential towards improving health outcomes in adults living with diabetes. The intervention improved glycemic level in diabetics, with an estimated 0.1% reduction in their glycosylated hemoglobin values in the treatment group (12). However, since the intervention was only targeted at middle-class working people, the findings would be difficult to extend to community level. In another intervention by Balagopal and colleagues in India, a significant reduction in blood glucose level was observed among the participants. Unfortunately, the study did not include a control group and lacked details

about the nature of community health workers(13). This reflects the need for more operational research in community health worker population approaches to reducing blood sugar among persons with type 2 diabetes.

14.3 Conceptual framework

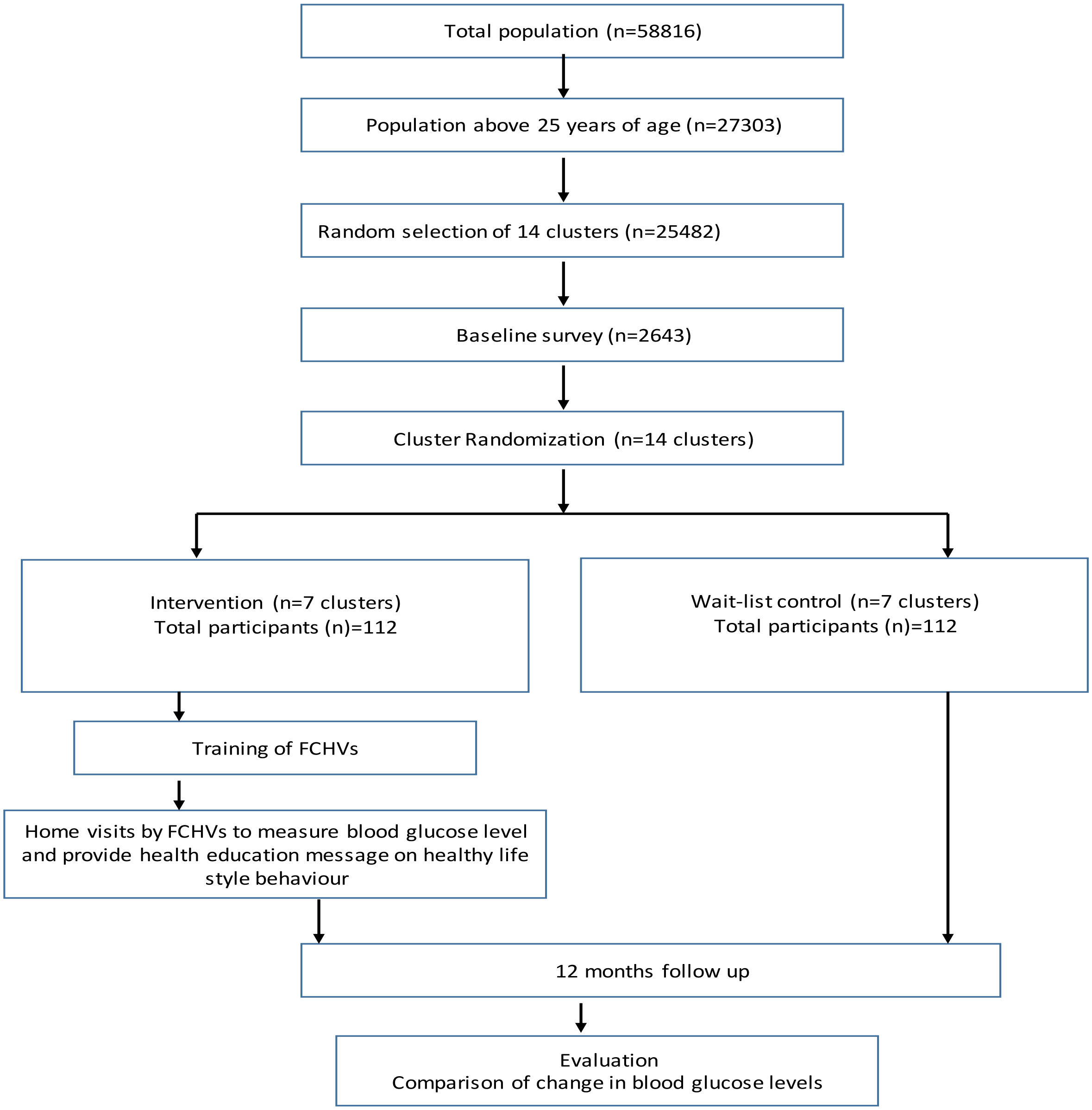

14.4 Research Objectives / purpose / aim of the study:

General

To explore the potential role of female community health volunteers for diabetes management at Lekhnath Municipality of Nepal

Specific

To evaluate the effect of a family-based home health educational intervention administered by female community health volunteers on reduction of blood glucose levels among diabetes individuals

15. Research Design and Methodology

Research Method

Qualitative ☐ Quantitative ☑ Combined ☐

Study Variables:

Primary outcome: Measures of glycemic control (Mean fasting blood glucose levels)

Secondary outcomes: Body Mass Index, blood pressure, alcohol use, tobacco use, diet intake, knowledge, quality of life, depression scores, physical activity, social support, and sedentary behaviors.

Type of Study (Specify):

Community-based cluster-randomized controlled trial

Study Site and Its Justification:

The study will take place in the Lekhnath Municipality, a semi-urban area of the Western Region of Nepal (Figure 1). The municipality has a total population of 58,816 in the census carried out in 2011 with 14,937 households and is administratively divided into 15 smaller units called *wards*. Each ward will be considered as 1 cluster. The municipality has a literacy rate of 85%, life expectancy of 59.7 years, and sex distribution of 31,951 females and 26,865 males(14). According to the Kaski District Public Health Office, there were 123 FCHVs in the municipality in 2013. No previous population-based interventions for diabetes at the population level have been carried out in the past. The epidemiological transition in the Western Region is more advanced than elsewhere in Nepal; indeed, it has the highest proportion of those with severe major risk factors for non-communicable diseases(15).

Study Population (Specify):

The study population is diabetes patients who will be identified by screening.

Study Unit:

Wards (Clusters)

Sampling Methods / Techniques (Specify)

The COBIN project has a population framework of all eligible participants prepared from the Voter List of the Election Commission. A systematic random sampling will be conducted to select participants in the baseline survey. If there is more than one participant from the same household eligible to participate in the study at the time of data collection, the *Kish* method will be adopted to select the participant. The same method will be applied for recruiting participants in the intervention phase.

The Lekhnath Municipality has 15 administrative units called wards (clusters). Out of 15 such wards, 1 ward was excluded by using simple random sampling before the randomization. Randomization was done for the clusters dividing 7 as intervention and 7 as control. After that, respondents who participated in the baseline survey will be the participants of the study.

Data will be collected by specially trained research assistants with a health professional background. Data about sociodemographic factors, lifestyle and prior illness will be collected by interviewing participants using STEPS questionnaires. Anthropometric data (weight, height, waist circumference) and blood pressure will be measured by the trained research assistants with a health professional background, height by a flexible non-stretchable measuring tape, weight by digital weighing machine, waist circumference by centimeter scale and blood pressure by digital sphygmomanometer.

Fasting blood glucose for the participants will be estimated by using a standardized digital glucometer (Infopia Co., Korea), using the capillary finger prick method. On a pre-informed date, fasting blood glucose estimation will be done. (Fasting being defined as no caloric intake for at least eight hours). Participants will be reminded by telephone for fasting glucose testing a day before the test. A person will be considered to be having diabetes if he/she will have fasting capillary blood glucose ≥7.0 mmol/l.

The baseline survey will be performed in both the intervention and wait-list control clusters. The survey tool will be adopted from the WHO STEPWISE Package, which includes physical measurement (height, weight), socio-demographic information (age, sex, family size, occupation, income, and education), and life style factors (current exposure to tobacco and alcohol, diet and physical activity). Participants will be asked to provide a written consent to have measured via a finger stick blood sample and Glucometer. The record of FCHV visit will be maintained by a supervisor. The same methodology will be used to conduct the follow-up survey after one year in both intervention and wait-list control clusters. Knowledge and skills of FCHVs will also be assessed.

A 12-month family-based life style intervention will be administered through the help of FCHVs. The framework will be based on the Health Belief Model. According to this model, the life style intervention program will include objectives based on individual perceptions (perceived susceptibility, perceived severity) and likelihood of action (perceived benefits, perceived barriers, cues to action and self-efficacy) of diabetes prevention. The intervention component will be developed by a panel consisting of health education experts, diabetologists, health workers and a diabetes patient. FCHVs will receive a seven-day training package after randomization highlighting: a) Introduction to the non-communicable diseases/diabetes; b) identifying populations at risk using a checklist including physical inactivity, family history, smoking and tobacco use, and alcohol consumption; c) screening techniques of blood glucose level, body mass index, blood pressure , and other cardio-vascular diseases (CVDs), as well as referral for those who have poor glycemic scores; d) providing health education (type 2 diabetes, CVDs, their risk factors, meal planning, exercise training activity, maintaining or reducing body weight, smoking cessation and personal hygiene; e) recording, reporting and follow-up. Training materials will be developed by involving local major stakeholders. The developed materials will be pretested with the FCHVs of the Lekhnath Municipality. On average, one volunteer will have to visit 25 households 3 times a year and provide counseling and measure blood glucose levels of selected respondents. FCHVs will also receive health education materials (poster, pamphlet), test strips, glucometers, tape, digital sphygmomanometer, and a recording register during training sessions.

The intervention to wards in the wait-list control will be delayed for 12 months. The participants allocated to the control group will be put on a waiting list and offered to participate in the intervention only after completion of the 12 months' assessment of the intervention group.

The same methodology will be used to conduct the follow-up survey after one year in both the intervention and wait-list control clusters. i.e., a follow-up survey will be carried out through trained professional health workers such as nurses and health assistants.

Sample size (with justification):

Sample size calculation

Sample size for baseline survey is calculated for 95% confidence interval (z=1.96), based on the following assumptions: margin of error (alpha) =5%, estimated prevalence of diabetes= 9.5% (16), design effect=2 and anticipated response rate=80 %. We are only including age group ≥25 years, so we will have 4 age groups namely 25-34 years, 35-44 years, 45-54 years, 55-64 years for each sex (total strata=8). This will result in a sample size=2643. Sample size is calculated based on the method suggested by the WHO STEPWISE Approach(17). According to the STEPS sample size calculator, the margin of error of 0.05 is suggested in a baseline survey for the expected prevalence of 10% or greater. In addition, a smaller margin of error of 0.02 or 0.01 is considered appropriate when the expected prevalence is lower than 10%. In this study, the anticipated prevalence was 9.5%, which is very close to 10% and this being an assumption, the margin of error of 0.05 was included.

Sample size calculation for diabetic populations

A lifestyle intervention study in a primary health care setting showed a mean reduction in fasting plasma glucose level by greater than 1 mmol/L after one year intervention in the diabetic population (18). Based on the standard deviation of mean reduction of fasting plasma glucose in the intervention arm of 2.1 (18), considering intraclass correlation of 0.01 and the design effect of 1.1 and 80% power into consideration, we will need 7 clusters with 11 individuals per intervention arm. To allow for the predicted dropout rate of 30%, the sample size was increased to 16 in each group i.e. 112 individuals per intervention group and the total sample size of the study will be 224.

Data Collection Technique / Methods (Specify):

The baseline survey will be performed in both the intervention and wait-list control clusters. The survey tool will be adopted from the WHO STEPWISE Package, which includes physical measurement (height, weight), socio-demographic information (age, sex, family size, occupation, income, and education), and life style factors (current exposure to tobacco and alcohol, diet and physical activity). Participants will be asked to provide a written consent to have measured via a finger stick blood sample and Glucometer.

The record of FCHV visit will be maintained by a supervisor. The same methodology will be used to conduct the follow-up survey after one year in both intervention and wait-list control clusters.

Data Collection Tools: (please attached in annex)

Questionnaires

Pre-testing the Data Collection Tools (if applicable):

Pre-testing of data collection tools will be conducted in the Lekhnath Municipality

Validity and Reliability of the Study Tools:

WHO Stepwise Survey tool will be taken as a reference material for baseline and follow-up survey which will be adopted from Nepal Health Research Council's survey tool. The training materials will be developed based on the already available materials from WHO and other similar organizations.

Potential Biases (if applicable):

There may be possibility of a selection bias in this study due to high loss to follow-up or failure to use intention to treat analysis. Moreover, selection bias can also be introduced through participant recruitment as our study will recruit participants after the clusters have been randomly allocated.

In the same way recruitment bias could occur in this study due to poor allocation concealment. If the allocation could not be concealed from the people doing the recruiting, there may be danger of recruitment bias.

Another important bias that could occur is the contamination bias. For instance, the subjects in the control group can be inadvertently exposed to the intervention group.

However, the risk of contamination will be minimized by randomization at the level of clusters. In order to reduce the possibility of contamination, we also have plans to collect every possible means of contamination between intervention and wait-list control participants during the follow-up survey, and, if these are found to be significant, we will make adjustments while estimating the effect.

Limitation of the Study:

16. Plan for Supervision and Monitoring:

The research team combines experience in epidemiology, global health, biostatistics, primary health care, type 2 diabetes, international research and health promotion. The supervisors have a long record of teaching and supervising at undergraduate, master and postgraduate levels, and they have the necessary expertise for this supervision obligation.

The PhD student will collect the primary data in Nepal, perform the analysis, and draft the three planned papers. The week to week supervision will be handled by Per Kallestrup and Annelli Sandbæk, and during fieldwork by Abhinav Vaidya and Dinesh Neupane.

17. Plan for Data Management and Analysis:

All analyses will be intention to treat, i.e., without regard to the compliance of individuals within their allocated study arm and with clustering-effect. At first, the baseline and follow-up characteristics will be compared to detect significant differences using appropriate tests such as student t-tests for normally distributed variables and chi-squared tests for categorical variables. As randomization will be at the ward (cluster) level in the study, random-effects model will be used to account for clustering effect. In addition, sensitivity of findings will also be tested by completing a per protocol analysis with the complete data set. In the event that randomization does not control for differences between the intervention and wait-list control cluster on baseline characteristics, we will statistically control for those differences in subsequent analysis of program effects. The final outcome will be modeled by using multiple linear regression analysis. All statistical analyses will be carried out in STATA, version 12. Two-sided p values will be reported with a statistical significance level of <0.05.

18. Expected Outcome of the Research:

The perspective of this study is to develop and implement, in collaboration with the community people, community-based, culturally sensitive diabetes prevention and control programs. Monitoring blood glucose with point-of-care testing at home can diagnose as well as monitor how well diabetes is controlled. The project thus aims to reduce morbidity and mortality from type 2 diabetes through regular screening of blood glucose levels by mobilizing community health workers and establishing a referral system. Reducing blood glucose level may lead to a big impact on reducing morbidity and mortality from type 2 diabetes. It will further increase our knowledge on how to control blood sugar by mobilizing community health volunteers. Hence, the research output has the potential to bring immediate benefits to address non-communicable disease. At the same time, the project is highly interdisciplinary and combines culturally appropriate health education and health promotion approaches to diabetes education, engages and trains locally available FCHVs for non-communicable disease prevention including diabetes, as well as establishes local coalition whose focus is on increasing awareness and promoting healthy lifestyle changes. Involving local volunteers will help to address social, cultural, political, and economic systems to change health behaviors and outcomes of the community, which ultimately supports to place a high priority on transferring findings into new practices and policies. The assessment of FCHVs' skill may help to develop a policy that can be scaled-up to a national level. If the health promotion package is found effective, the approach can be easily adopted into the existing health care delivery system in Nepal. The research output can bring immediate benefits to the individuals of the intervention area as well as contribute to the development of a national diabetes management plan. Collaboration with the Ministry of Health and Population of Nepal is ensured already at the planning phase in order to stream-line the potential beneficial results of the project into policy with these regards. It is anticipated that the program can act as a feasible and affordable tool for evidence-based integrated care for improvement of diabetes management and outcomes in Nepal as well as in low- and middle-income countries elsewhere.

19. Plan for Dissemination of Research Results:

The Principal investigator will use this project as part of his PhD thesis. So, it will be disseminated in his unit at the university. Three manuscripts will be prepared together with co-investigators to publish in international peer-reviewed journals. In addition to this, the research will be disseminated at district level and national level by organizing workshops and seminars in collaboration with Ministry of Health and Population. Further, findings will also be disseminated through participation in several national and international conferences.

20. Plan for Utilization of the Research Findings (optional):

How is the research project going to strengthen the research capability of the host institution: Nepali Researcher (if submitted from abroad):

The principal applicant is from Nepal. As a PhD student he will strengthen the research capability of himself through close supervision and guidance of the experts from Nepal and Denmark. He will engage in collaboration and mutual capacity-building with Nepali research colleagues of national institutions

21. Work Plan *(should include duration of study, tentative date of starting the project and work schedule / Gantt chart):*

Activities/month	2016												2017												2018								
	1	2	3	4	5	6	7	8	9	10	11	12	1	2	3	4	5	6	7	8	9	10	11	12	1	2	3	4	5	6	7	8	
Detail plan of protocol	■	■	■	■																													
Literature/Fund search		■	■	■	■	■	■																										
Office Set Up				■	■	■	■	■																									
Preparation of baseline survey									■	■																							
Baseline survey											■	■																					
Data analysis of baseline survey													■	■																			
Design & test training materials													■																				
Training to FCHVs														■																			
Intervention															■	■	■	■	■	■	■	■	■	■	■	■							
Follow up survey																											■	■	■				
Dissemination															■															■			
Report writing/publication								■								■															■	■	■
Monitoring and supervision									■	■	■	■	■	■	■	■	■	■	■	■	■	■	■	■	■	■	■	■	■	■	■	■	■
Project management	■	■	■	■	■	■	■	■	■	■	■	■	■	■	■	■	■	■	■	■	■	■	■	■	■	■	■	■	■	■	■	■	■

References:

1. Shaw JE, Sicree RA, Zimmet PZ. Global estimates of the prevalence of diabetes for 2010 and 2030. *Diabetes Res Clin Pr.* 2010;87(1):4-14.
2. WHO. Diabetes mellitus: fact sheets (Online). WHO Publ. 2015. Available from http://www.who.int/mediacentre/factsheets/fs138/en/. Retrieved on 08.02.2015.
3. Hussain A, Claussen B, Ramachandran A, Williams R. Prevention of type 2 diabetes: a review. *Diabetes Res Clin Pr.* 2007;76(3):317-326.
4. International Diabetes Federation. Diabetes atlas sixth edition poster update 2014. 2014. Brussels, Belgium.
5. Gyawali B, Sharma R, Neupane D, Mishra SR, van Teijlingen E, Kallestrup P. Prevalence of type 2 diabetes in Nepal: a systematic review and meta-analysis from 2000 to 2014. Glob Health Action. 2015;8:29088.
6. International Diabetes Federation. Diabetes atlas sixth edition poster update 2014. 2014. Brussels, Belgium.
7. Eriksson J, Lindström J, Tuomilehto J. Potential for the prevention of type 2 diabetes. *Br Med Bull.* 2001;60(1):183-199.
8. WHO. World Health Organization 2008-2013 action plan for the global strategy for the prevention and control of noncommunicable diseases. Geneva, Switzerland: WHO Publ. 2008.
9. Kosaka K, Noda M, Kuzuya T. Prevention of type 2 diabetes by lifestyle intervention: a Japanese trial in IGT males.*Diabetes Res Clin Pr.* 2005;67(2):152-162.
10. Pan XR, Li GW, Hu YH, et al. Effects of diet and exercise in preventing NIDDM in people with impaired glucose tolerance. The Da Qing IGT and Diabetes Study. *Diabetes Care.*1997;20(4):537-544.
11. Snehalatha C, Mary S, Joshi VV, Ramachandran A. Beneficial effects of strategies for primary prevention of diabetes on cardiovascular risk factors: results of the Indian Diabetes Prevention Programme.*Diab Vasc Dis Res.*2008;5(1):25-29.
12. Ramachandran A, Snehalatha C, Mary S, Mukesh B, Bhaskar AD, Vijay V. The Indian Diabetes Prevention Programme shows that lifestyle modification and metformin prevent type 2 diabetes in Asian Indian subjects with impaired glucose tolerance (IDPP-1). Diabetologia. 2006;49(2):289-97.
13. Balagopal P, Kamalamma N, Patel TG, Misra R. A community-based diabetes prevention and management education program in a rural village in India. *Diabetes Care.*2008;31(6):1097-1104.
14. WSP. Lekhnath Municipality (online). Available from: http://lekhnathwatersupply.weebly.com/. Retrieved on 29.01.2015.
15. Bhandari G, Angdembe M, Dhimal M, Neupane S, Bhusal C. State of non-communicable diseases in Nepal. *BMC Public health.* 2014;14(1):23.
16. Ono K, Limbu YR, Rai SK, et al. The prevalence of type 2 diabetes mellitus and impaired fasting glucose in semi-urban population of Nepal. *Nepal Med Coll J.* 2007;9(3):154-156.
17. WHO. Chronic diseases and health promotion. WHO Publ. 2014; Available from: http://www.who.int/chp/steps/resources/en/. Retrieved on 18.02.2015
18. Chao J, Wang Y, Xu H, et al. The effect of community-based health management on the health of the elderly: a randomized controlled trial from China. BMC Health Serv Res. 2012;12:449.

Part – IV

Ethical Consideration

22. Regarding the human participants:

Are human participants required in this research? If yes, provide justification.

[√] Yes (*provide justification*) No [ ]

How many participants are required for the research? Explain.

2643

What is the frequency of the participant's involvement in the research? Explain.

2

Clearly indicate the participant's responsibilities in the research. What is expected of the research participants during the research?

Participants will provide information and receive health information package in intervention VDCs

Are vulnerable members of the population required for this research? If yes, provide justification.

No

Are there any risks involved for the participants? If yes, identify clearly what are the expected risks for the human participants in the research and provide a justification for these risks.

No

Are there any benefits involved for the participants? If yes, identify clearly what are the expected benefits for the participants.

For the participants in the intervention VDCs, they will get an opportunity to measure their blood glucose level, blood pressure and receive health education package from FCHVs.

23. Informed Consent Form / Ethical Issues:

Statements required in the Informed Consent Form include:

A statement that the human participants can withdraw from the study at any time without giving reason and without fear. State clearly how the participants can opt out the study.

A statement guaranteeing the confidentiality of the research participants.

If required, a statement on any compensation that might be given to the research participant and or their community.

A statement indicating that the participants has understood all the information in the consent form and is willing to volunteer / participate in the research.

Signature space for the research participants, a witness, and the date.

(Informed Consent form should be submitted in English and in the language appropriate to the research participants)

Obtaining the Consent
How informed consent is obtained from the research participants?

[ ] Verbal [√] Written

Please indicate who is responsible for obtaining informed consent from the participants in this research study?

……………………………………..……………………………

Is there anything being withheld from the research participants at the time the informed consent is being sought?

If yes, explain

……………………………………………………………………………

Is the research sensitive to the Nepali culture and the social values?

Yes ☐ No ☑ Explain.

……………………………………………………………………………

Is health insurance *(if applicable)* being made available to the research participants? If yes, please provide the necessary insurance data.

……………………………………………………………………………

(Include in consent form)

24. Regarding Clinical Trial:

In case of a clinical trial address the following:

The trial treatment

……………………………………………………………………………

A detailed explanation of the trial procedures including all invasive procedures.

……………………………………………………………………………

The potential or direct benefits (if any) for the research participants.

……………………………………………………………………………

Alternative procedure(s) or treatment(s) that may be available.

……………………………………………………………………………

The risks, discomforts, and inconveniences associated with the study

Infection and pain at blood sampling site

……………………………………………………………………………

Provisions for management of any adverse reactions

To avoid these risks, a well-trained person will be hired to take the blood sample and hand hygiene will be performed

……………………………………………………………………………

The provisions of insurance coverage for any permanent disability or death caused directly by the investigational treatment or procedure.

……………………………………………………………………………

The provision of including the name and address, including telephone numbers of person to be contacted in case of adverse events or for any information related to the trial.

……………………………………………………………………………

Is there going to be a transfer of any biological materials from the country? Explain.

……………………………………………………………………………

Is there a Data Safety Monitoring Board?
If Yes, Mention

……………………………………………………………………………

Is this trail internationally registered?

……………………………………..

Part – V

ACCEPTANCE OF GENERAL CONDITIONS AND DECLARATION BY THE PRINCIPAL INVESTIGATOR

I hereby certify that the above mentioned statements are true, I have read and understood the regulation of the Nepal Health Research Council (NHRC) on the approval of research proposal and will act in conformity with the said regulation in all respects.

If the research is terminated, for any reason, I will notify NHRC of this decision and provide the reasons for such actions. I will provide NHRC with a written notice upon the completion of the research as well as a final summary/full report of the research study. If I publish the results in a journal, I shall acknowledge the NHRC and shall provide the Council with three copies of any such articles.

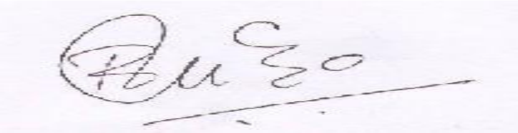

…………………………

Signature of Applicant

Date: …August 20, 2016………………

INFORMED CONSENT:

- Describe the manner in which informed consent will be obtained.

- Indicate what kind of consent (e.g. parental, child, adult, etc) will be used.

- If the subjects are children/adolescents ages 7-18 years, an Assent Form must be included with the IRB application. The signed Assent Form along with the Parental/Guardian Consent Form must be retained on file for at least three years after completion of the research project.

- If prisoners / pregnant women, or fetuses are to be included in the research sample, it is likely that a full IRB review will be required and additional human subjects' protections will be expected.

- If the subjects do not read or comprehend English, you must provide a consent form in their language as well as in English for IRB review and approval.

- If you are requesting a waiver of written consent (i.e. a signature on an informed consent form) from the subjects, you MUST justify this request by providing an explanation of why obtaining written consent would add additional risk to the subjects and your alternative provisions for informing them about the study.

- If consent documents from another site will be used, you will have to indicate this and provide a copy of the authorized consent document and IRB approval with your application.

- You will have to provide any other relevant information if necessary. Please be aware that the PI is legally required to retain all signed Informed Consent forms for at least three years after the project terminates

- The Informed Consent form must be written at a level that the subjects will understand. Please use simple language, and avoid clinical jargon.

- Attach a copy of the written informed consent form (assent or parental consent where applicable). Consent documents MUST be in format requested. See examples on line.

- If the study uses database or archival data the use of informed consent is not applicable.

CONFIDENTIALITY OF DATA: ***Confidentiality of data MUST be address for all studies.***

- Indicate the extent to which confidentiality of records identifying subjects will be maintained.

- Describe the storage and disposal of information where applicable.

Check List

For all applicants

1. Covering letter addressed to the Member secretary indicating the submission of the approval of proposal.
2. Proposal will only be accepted if submitted in NHRC format.
3. Both printed and electronic version of the proposal should be submitted.
4. Curriculum Vitae of the Principal Investigator & Co-Principal Investigator of the study team should be submitted.
5. If the Principal Investigator is a non Nepali citizen, at least one Co-investigator should be a Nepali citizen.
6. Submission of the application processing fee to NHRC.(According to NHRC rules and regulations)
7. Source of funding for the proposed project.
8. The proposal should have institutional ethical clearance from his/her own country if submitted from academic and related institution.
9. If the research study is to be conducted in any hospitals/organization or institution/community, a letter of approval from the related hospital/organization or institution/district authority should be provided.
10. Consent form should be in Nepali & local language (if necessary).
11. Data collection tools should be in Nepali & local language (if necessary) including interview guideline, observation checklist, questionnaires etc.
12. Style of referencing should be in Harvard style.
13. List of abbreviations / acronyms should be provided.

For students' applicants

1. Approval letter from concern Institute/University.
2. Recommendation letter from Academic Supervisor.

Processing Fee

Researcher has to pay the processing fee as per the rules and regulations of NHRC.
