# Supplementary material for: Effectiveness of a Female Community Health Volunteer–Delivered Intervention in Reducing Blood Glucose Among Adults With Type 2 Diabetes: An Open-Label, Cluster Randomized Clinical Trial
Source: JAMA Netw Open. 2021 Feb 1;4(2):e2035799. doi: 10.1001/jamanetworkopen.2020.35799 (PMC7851734; doi:10.1001/jamanetworkopen.2020.35799)
Supplement: Supplement 2. — eFigure. Recording Register Example eTable 1. Supervision Checklist Example eTable 2. Baseline Characteristics of the Per Protocol Sample eTable 3. Imputation Analysis Using Intention-to-Treat Principles of Changes in Primary and Secondary Outcomes at 12 Months [file jamanetwopen-e2035799-s002.pdf]

Supplemental Online Content

Gyawali B, Sharma R, Mishra SR, et al. Effectiveness of a female community health volunteer–delivered intervention in reducing blood glucose among adults with type 2 diabetes: an open-label, cluster randomized clinical trial. *JAMA Netw Open*. 2021;4(2):e2035799. doi:10.1001/jamanetworkopen.2020.35799

eFigure. Recording Register Example
eTable 1. Supervision Checklist Example
eTable 2. Baseline Characteristics of the Per Protocol Sample
eTable 3. Imputation Analysis Using Intention-to-Treat Principles of Changes in Primary and Secondary Outcomes at 12 Months

This supplemental material has been provided by the authors to give readers additional information about their work.

© 2021 Gyawali B et al. *JAMA Network Open.*

eFigure. Recording Register Example

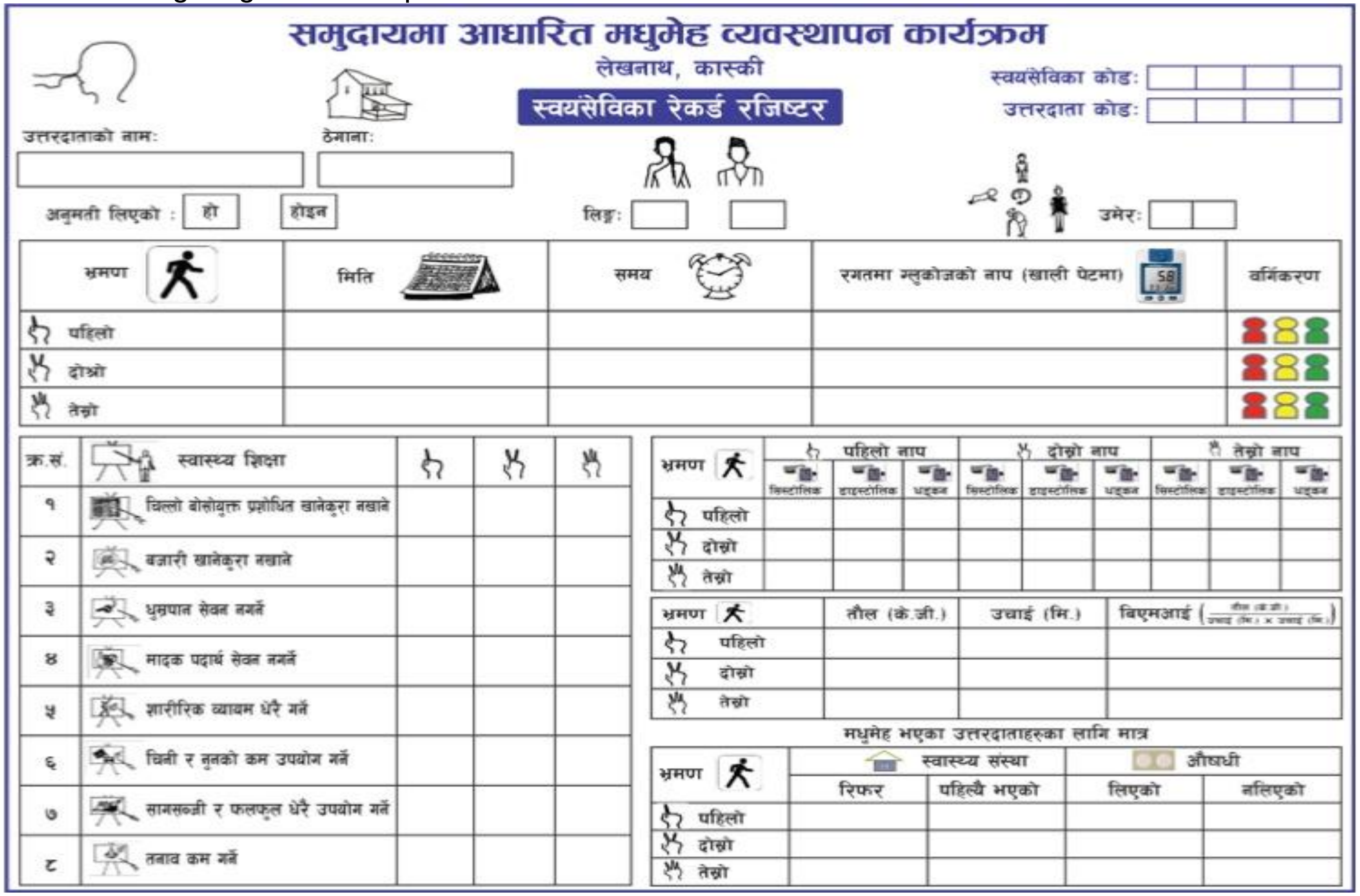

समुदायमा आधारित मधुमेह व्यवस्थापन कार्यक्रम

लेखनाथ, कास्की

स्वयंसेविका रेकर्ड रजिष्टर

स्वयंसेविका कोड:

उत्तरदाता कोड:

उत्तरदाताको नाम:

ठेगाना:

अनुमती लिएको : हो / होइन

लिङ्ग:

उमेर:

भ्रमण	मिति	समय	रगतमा ग्लुकोजको नाप (खाली पेटमा)	वर्गिकरण
पहिलो				
दोस्रो				
तेस्रो				

क्र.सं.	स्वास्थ्य शिक्षा			
१	चिल्लो बोसोयुक्त प्रशोधित खानेकुरा नखाने			
२	बजारी खानेकुरा नखाने			
३	धुम्रपान सेवन नगर्ने			
४	मादक पदार्थ सेवन नगर्ने			
५	शारीरिक व्यायम धेरै गर्ने			
६	चिनी र नुनको कम उपयोग गर्ने			
७	सागसब्जी र फलफुल धेरै उपयोग गर्ने			
८	तनाव कम गर्ने			

भ्रमण	पहिलो नाप			दोस्रो नाप			तेस्रो नाप		
	सिस्टोलिक	डाइस्टोलिक	धड्कन	सिस्टोलिक	डाइस्टोलिक	धड्कन	सिस्टोलिक	डाइस्टोलिक	धड्कन
पहिलो									
दोस्रो									
तेस्रो									

भ्रमण	तौल (के.जी.)	उचाई (मि.)	बिएमआई (तौल (के.जी.) / उचाई (मि.) x उचाई (मि.))
पहिलो			
दोस्रो			
तेस्रो			

मधुमेह भएका उत्तरदाताहरुका लागि मात्र

भ्रमण	स्वास्थ्य संस्था		औषधी	
	रिफर	पहिल्यै भएको	लिएको	नलिएको
पहिलो				
दोस्रो				
तेस्रो				

Recording register used by FCHVs to record dates, times and activities.

© 2021 Gyawali B et al. *JAMA Network Open.*

eTable 1. Supervision Checklist Example

eTable 1. **Supervision checklist of FCHV Household Visits**

Community-Based Management of Diabetes Project in Nepal

Name of FCHV:
Ward No:
Date:

S. No	Indicators	Not done	Poorly done	Well done	Remarks
1	Greeted participants				
2	Introduced herself and explained the purpose of visit				
3	Measured blood glucose using glucometer accurately				
4	Measured blood pressure using digital monitor accurately				
5	Measured height using standard stature scales accurately				
6	Measured weight using digital personal scales accurately				
7	Provided counseling and health promotion message on major risk factors and medication compliance				
8	Used relevant information/education/communication materials				
9	Appropriately recorded information in the recording register				
10	Referral of participants				
11	Gave follow-up appointment				
12	Thanked participants				
13	Submitted quarterly reports to the field supervisor				
14	Attended quarterly meetings at the field office				

Overall positive comments: ____________________

Suggestions for improvement: ____________________

Any follow-up required: ____________________

© 2021 Gyawali B et al. *JAMA Network Open.*

eTable 2. Baseline Characteristics of the Per Protocol Sample

**Variables**	**Intervention group (N=107)**	**Control group (N=105)**
Cluster level		
Wards	7	7
FCHVs	20	0
Individual level		
Participants in clusters	107	105
Socio-demographic characteristics		
Age (years)	51.87 ± 8.11	52.73 ± 8.58
Sex		
Male	51 (47.6)	41 (39.1)
Female	56 (52.4)	64 (60.9)
Education		
Low	60 (56.1)	60 (57.1)
Medium	43 (40.2)	38 (36.2)
High	4 (3.7)	7 (6.7)
Occupation		
Agriculture	42 (39.3)	31 (29.5)
Employee	18 (16.9)	18 (17.1)
Housemaker	29 (27.1)	40 (38.1)
Labour	3 (2.8)	2 (1.9)
Others	15 (14.0)	14 (13.4)
Monthly household income (Nepalese Rupees)	26911.21 ± 21214.40	32933.33 ± 29579.83
Marital status		
Married	9 (8.4)	11 (10.5)
Unmarried	98 (91.6)	94 (89.5)
Clinical characteristics		
Weight (kg)	66.33 ± 11.32	66.50 ± 11.46
BMI (kg/m^2)[a]	26.74 ± 4.00	27.12 ± 4.01
Systolic blood pressure (mm Hg)	133.55 ± 15.36	132.19 ± 18.60
Diastolic blood pressure (mm Hg)	85.98 ± 7.99	85.29 ± 11.86
Biochemical characteristics		
Fasting blood glucose (mg/dl)	157.14 ± 42.66	153.18 ± 43.38
Behavioural characteristics		
Current smoking[b]	30 (28.0)	24 (22.9)
Harmful alcohol consumption[c]	18 (16.8)	11 (10.5)
Low physical activity[d]	20 (18.7)	19 (18.1)
Low fruit and vegetables intake[e]	102 (95.3)	102 (97.1)
Medical history		
Family history of diabetes	36 (33.6)	49 (46.7)
Receiving antihyperglyecmic medications	63 (58.9)	43 (40.9)

Data are n (%), mean ± SD.

Abbreviations: FCHVs, Female Community Health Workers; SD, standard deviation; BMI, Body Mass Index.

All data are unadjusted.

Difference between intervention and control group determined by ANOVA test, Chi-squared or Fishers-exacts tests.

[a]BMI was computed as weight in kilogram (kg) divided by the height in meters squared (m^2).

© 2021 Gyawali B et al. *JAMA Network Open.*

[b]Current smoking was defined as those who smoked cigarettes, tobacco variants (bidi, kankat or hukka) or those using other forms of smokeless tobacco daily.
[c]Harmful alcohol consumption was determined by asking the number of standard drinks consumed in the last 30 days. Harmful alcohol was defined as drinking 15 or more standard units of alcohol a week for men and eight or more standard units a week for women.
[d]Low physical activity was defined as less than 3000 metabolic equivalents of tasks (METs) of vigorous or moderate activity per week.
[e]Participants consuming fewer than five servings of fruits or vegetables a week were categorized as low fruits and vegetables intake. One serving of vegetables was defined as one cup of raw green leafy vegetables, a half cup of other vegetables (cooked or chopped raw) or a half cup of vegetable juice. One serving of fruit was defined as one medium-sized piece of fruit, a half cup of chopped, cooked, canned fruit or a half cup of non-artificially flavored fruit juice.

© 2021 Gyawali B et al. *JAMA Network Open.*

eTable 3. Imputation Analysis Using Intention-to-Treat Principles of Changes in Primary and Secondary Outcomes at 12 Months

Outcomes	Intervention Group (N=127)	Control Group (N=117)	Intervention Effect	P value§
Fasting blood glucose–mg/dl#				
At baseline	158.40 ± 45.54	153.54 ± 43.38		
At 12 months	133.74 ± 43.56	160.38 ± 57.06		
Change (95% CI)	-24.66 (-35.64, -13.50)	6.84 (-6.12, 19.80)	-27.99 (-37.71, -18.26) ¶	<0.001
Systolic blood pressure–mm Hg				
At baseline	134.14 ± 16.73	133.30 ± 20.68		
At 12 months	125.57 ± 16.21	130.05 ± 17.51		
Change (95% CI)	-8.57 (-12.64, -4.49)	-3.25 (-8.18, 1.68)	-6.14 (-10.20, -2.08) ¶	0.003
Diastolic blood pressure–mm Hg				
At baseline	86.00 ± 8.99	85.51 ± 12.25		
At 12 months	80.94 ± 9.68	81.61 ± 10.04		
Change	-5.06 (-7.36, 1.57)	-3.90 (-6.78, -1.01)	-2.25 (-5.60, 1.08) ¶	0.18
BMI–kg/m^2				
At baseline	26.39 ± 3.96	26.84 ± 4.11		
At 12 months	26.37 ± 4.16	26.97 ± 4.37		
Change	-0.02 (-1.02, 0.98)	0.13 (-0.94, 1.20)	-0.36 (-1.21, 0.47) ¶	0.39
Low physical activity				
At baseline	23 (18.1)	23 (19.6)		
At 12 months	13 (10.2)	15 (12.8)		
Change	1.7 (0.9, 3.3)	1.5 (0.8, 2.7)	1.05 (0.44, 2.51)‡	0.89
Harmful alcohol consumption				
At baseline	21 (16.54)	13 (11.11)		

© 2021 Gyawali B et al. *JAMA Network Open.*

At 12 months	15 (11.81)	12 (10.26)		
Change	1.4 (0.8, 2.6)	1.1 (0.5, 2.2)	1.18 (0.59, 2.35)‡	0.62
Current smoking				
At baseline	38 (29.9)	27 (23.0)		
At 12 months	40 (31.5)	23 (19.6)		
Change	0.9 (0.6, 1.3)	1.1 (0.7, 1.9)	1.55 (0.63, 3.85)‡	0.33
Low fruit and vegetable intake				
At baseline	122 (96.0)	114 (97.4)		
At 12 months	122 (96.0)	115 (98.2)		
Change	1.00 (0.9, 1.1)	1.0 (0.9, 1.0)	0.36 (0.06, 2.19)‡	0.27
Receiving antihyperglycemic medications				
At baseline	73 (57.4)	47 (40.2)		
At 12 months	75 (59.1)	59 (50.4)		
Change	0.9 (0.7, 1.2)	0.8 (0.6, 1.1)	1.84 (1.06, 3.18)‡	0.03

* Plus-minus values are means changes and 95% confidence interval.

# The pre-specified primary outcome was the mean change in fasting blood glucose in intervention and control group from baseline to follow-up. Intra-class Correlation Coefficient from the linear mixed effects model for change in fasting blood glucose was <0.001.

§ For continuous outcomes, P values were calculated from linear mixed-effects models with random intercepts for wards and participants. The estimated intervention effect was controlled for age, sex, antihyperglycemic medication status, family history of diabetes, monthly household income and the baseline summary of the respective outcomes. For binary outcomes, P values were calculated from mixed-effects logistic regression analyses with random intercepts for wards and participants. The estimated intervention effect was controlled for age, sex, antihyperglycemic medication status, family history of diabetes, monthly household income and the baseline summary of the respective outcomes.

¶ Shown is the difference in mean change and 95% confidence interval.

‡ Shown is the relative risk and 95% confidence interval

© 2021 Gyawali B et al. *JAMA Network Open.*
