# Supplementary material for: Effectiveness of a Female Community Health Volunteer–Delivered Intervention in Reducing Blood Glucose Among Adults With Type 2 Diabetes: An Open-Label, Cluster Randomized Clinical Trial
Source: JAMA Netw Open. 2021 Feb 1;4(2):e2035799. doi: 10.1001/jamanetworkopen.2020.35799 (PMC7851734; doi:10.1001/jamanetworkopen.2020.35799)
Supplement: Supplement 3. — Data Sharing Statement [file jamanetwopen-e2035799-s003.pdf]

Data Sharing Statement

Gyawali. Effectiveness of a Female Community Health Volunteer-Delivered Intervention in Reducing Blood Glucose Among Adults With Type 2 Diabetes. *JAMA Netw Open*. Published February 01, 2021. doi:10.1001/jamanetworkopen.2020.35799

Data
Data available: Yes
Data types: Deidentified participant data
How to access data: bigyawali@sund.ku.dk
When available: With publication

Supporting Documents
Document types: Other (please specify)
Additional Information: Informed consent form, questionnaires
How to access documents: bigyawali@sund.ku.dk
When available: With publication

Additional Information
Who can access the data: anyone requesting the data
Types of analyses: for any purpose
Mechanisms of data availability: signed data access agreement
